# Wheat Germ Agglutinin (WGA): Its Nature, Biological Role, Significance in Human Nutrition, and Possibility to Be Used as Marker of Whole-Grain Status in Wheat-Based Foods

**DOI:** 10.3390/foods13182990

**Published:** 2024-09-21

**Authors:** Marina Carcea, Sahara Melloni, Valentina Narducci, Valeria Turfani

**Affiliations:** Research Centre for Food and Nutrition, Council for Agricultural Research and Economics (CREA), Via Ardeatina 546, 00178 Rome, Italy; sahara.melloni@crea.gov.it (S.M.); valentina.narducci@crea.gov.it (V.N.); valeria.turfani@crea.gov.it (V.T.)

**Keywords:** wheat germ agglutinin, lectins, whole-grain food, wholegraininess, biomarker, ELISA

## Abstract

The growing scientific evidence on the health benefits of whole-grain food consumption has promoted the manufacturing of a great number of products differing in quality and content of whole-grain components. This is particularly true for commercial wheat-based products where it is not always clear how much whole wheat is present considering that in many cases, they are manufactured from reconstituted mill streams and that there is not a standardised globally accepted definition and metrics to objectively evaluate whole-grain status. Attempts have been made to assess the level of “wholegraininess” in wheat products by measuring specific constituents that correlate with different wheat tissues, especially those that are expected to be found in a true whole-grain wheat product. Wheat germ agglutinin (WGA), a small lectin protein present exclusively in the wheat-germ tissues, has been indicated by several scientists as one of these constituents and after founding that its level changes depending on the amount of germ found in a wheat flour, it has been indicated as a biomarker of whole-grain status for wheat products. In this review, the biochemistry of WGA, its methods of detection, and current knowledge on its possibility to be practically utilized as a reliable marker are critically discussed.

## 1. Introduction

Wheat germ agglutinin (WGA) is a protein belonging to the lectins group, present in the wheat germ. The germ contains about 30% proteins and 10–15% lipids that are prone to oxidation and rancidity, and its polyphenol oxidase is prone to react with phenolic substances, leading to the blackening of flour colour and even affecting its shelf life.

Therefore, during wheat processing, wheat germ is often discarded as a by-product or used as feed [1] when it is not used for oil extraction [2]. WGA is localized in the embryonic axis of the wheat germ, while the scutellum contains less than a thousandth of it with respect to the embryonic axis [3].

When germ is not removed from the kernel, with roller milling it ends up mostly in the bran and middlings, whereas with stone milling it is mixed with the whole meal. The possible use of WGA as a biochemical marker to quantify the proportion of embryonic axis tissue in wheat (*Triticum* spp.) milling fractions or wheat-based products is a matter of interest with the growing increase in whole-grain product consumption. Whole-grain wheat intake is being promoted for its beneficial health effects with respect to refined products worldwide as scientific evidence from epidemiological and intervention studies accumulates [4]. Whole grains have, in fact, been associated with a reduced risk of cardiovascular diseases, type 2 diabetes, colorectal, pancreatic and gastric cancers, weight gain, and obesity [5].

This nutritional interest, coupled with sustainability issues related to the consumption of unrefined products, has boosted worldwide the market of whole-grain ingredients and foods, and the food industry has produced a great variety of different products, possessing different compositions and hence different nutritional qualities even if they are all labelled as “whole-grain” foods. Nowadays a whole-grain flour can, for example, be produced by recombining bran fractions and refined flour as it happens with roller milling or it can be obtained directly from the wheat grains as with stone milling [6,7], and a whole-grain food can be produced by adding different percentages of whole-grain flour.

Considering the above, the scientific community and the food industry realized the need to globally define in clear terms a whole-grain raw material and a whole-grain food product as a basis for dietary recommendations, food labelling, legislation, health claims, and comparisons of nutrition researchers where the studied food should be unequivocally known. Recently, the Whole Grain Initiative (WGI) developed and published generic consensus definitions for whole grains and whole-grain foods, which were ratified by the Cereals and Grains Association, the International Association for Cereal Grain and Technology (ICC), and the Healthgrain Forum [8]. These definitions state that whole grains as food ingredients shall consist of the intact or processed kernels after the removal of inedible parts such as the hull and husk and that all anatomical components, including the endosperm, germ, and bran, must be present in the same relative proportions as in the intact kernel, whereas a whole-grain food shall contain at least 50% whole-grain ingredients based on dry weight.

Therefore, according to the above definitions, a whole-grain ingredient or food must contain all of the bran parts, including the germ. In fact, the germ being nutritionally rich in proteins, lipids, vitamins, and bioactive substances can be considered a valuable component of a whole-grain product.

It is important to underline the fact that, at present, in various countries, regulations regarding the definition of wholegrain flour may differ. Some countries may permit the removal of certain parts of the grain, such as the germ, while still labelling the flour as “whole grain.” However, the specifics can vary widely.

For example, in the United States, the Food and Drug Administration (FDA) defines whole grain as consisting of the intact, ground, cracked, or flaked fruit of the grains whose principal components, the starchy endosperm, germ, and bran are present in the same relative proportions as they exist in the intact grain [9]. However, in practice, the FDA allows for small losses of bran and germ during processing, provided they do not result in a significant reduction in the dietary fibre, iron, and other nutrients.

In Canada, when wheat is milled, parts of the kernel are separated and then recombined to make whole wheat flour. Under the Food and Drug Regulations, up to 5% of the kernel can be removed to help reduce rancidity and prolong the shelf life of whole wheat flour. The portion of the kernel that is removed for this purpose contains much of the germ and some of the bran [10]. This has been recently demonstrated in durum wheat in a paper by Marzocchi et al. [11], where they studied specific lipid markers in by-products obtained from five different debranning levels (3, 6, 9, 12, and 15%).

Though there is no legally endorsed definition of whole grain and whole-grain products and foods at the European level, in some European countries, such as Italy, the current legislation distinguishes a wholegrain wheat flour from a refined flour depending on the levels of quality parameters such as ash and proteins.

Being able to detect the presence of the germ in a flour or a product was therefore suggested by some scientists as a means to check the adherence of that specific product to the definition of a whole-grain flour or product [3], and the quantification of the WGA being present in the germ has been suggested as a potential biochemical marker to assure consumers about the “wholegraininess” of the food itself [12]. Moreover, the amount of WGA in wheat foodstuffs may be measured by means of rapid methods such as ELISA or dot blot methods where selective antibodies are utilized to identify it [13,14].

Considering the current need in cereal science and technology to progress on this new topic of wholegraininess and on reliable ways to prove it in the interest of industry and consumers, this review was planned to report on the state of the art of knowledge on WGA, its nature, biological significance in the plant, role in human nutrition, applications, and in particular its possibility to be used as a reliable quality marker for whole-grain wheat foodstuffs. Data from the literature are also compared with unpublished data coming from experiments conducted in the research labs of the authors to understand whether we can already count on rapid WGA measurement as a quality test for whole-grain wheat foodstuffs or for foodstuffs containing wheat germ in general. Wheat germ is in fact a major by-product of the milling industry, which, if appropriately stabilized by fermentation [15], can produce a raw material with improved nutraceutical properties to be used in functional foods.

## 2. Chemical Nature, Biological Role, and Presence of WGA in Wheat

### 2.1. Lectins in Nature

Lectins are natural proteins that specifically recognize and reversibly bind various carbohydrates [16], widely distributed in seeds and vegetative parts of edible plant species where they are probably involved in the plant defence and other functions requiring recognition of a carbohydrate moiety [17,18,19]. They have been first defined as “carbohydrate-binding proteins of non-immune origin that agglutinate cells or precipitate polysaccharides or glycoconjugates” [16,20,21,22].

They were first isolated from plants in the late 19th century and have been receiving increasing attention since mid-20th century. To date, it is known that lectins can originate from all kinds of living things (algae, fungi, bacteria, animals, plants). Lectins with diverse origins, molecular structures, biochemical and biophysical properties, and biological functions are known, and new ones are still being discovered and described in the literature [19,23,24].

Even the definition of lectin has been gradually expanded to include, alongside proteins containing two or more carbohydrate-binding domains and thus capable to agglutinate cells and bind glycoconjugates (now called *hololectins*), also proteins with only one carbohydrate-binding domain (called *merolectins*) and proteins containing enzymatic domains altogether with carbohydrate-binding domains (called *chimerolectins*), until it has become “all plant proteins possessing at least one non-catalytic domain, which binds reversibly to a specific mono- or oligosaccharide” (for an excursus on lectin definition, see Peumans and Van Damme [25]).

Animal lectins are usually secreted from the cells, where they may participate in cell-cell interactions, recognition of immune defence systems, immunoregulation, and prohibition of autoimmunity [26,27,28].

### 2.2. Molecular Structure and Binding Specificity of WGA

**Figure 1:**
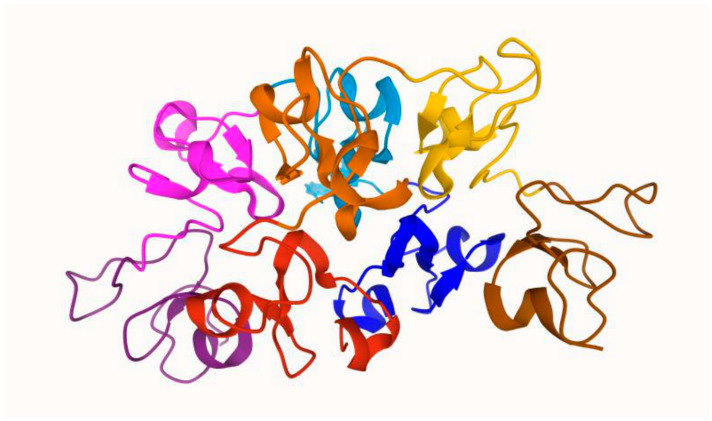
Dimeric structure of the wheat germ agglutinin 3 (WGA isoform n.3) (in Balčiūnaitė-Murzienė et al., 2021 [29]). Red, orange, yellow, and brown colors represent the subunits A, B, C, and D of the first protein, respectively. Blue, light blue, pink, and purple represent the subunits A, B, C, and D of the second protein, respectively. The sugar-binding site is located at the interface of both WGA monomers.

The WGA is a small plant lectin of the kind of *hololectins*. Structurally, it has been reported as a 17 kDa polypeptide chain [23] or as a homodimer of two 18.0 kDa polypeptide chains joined with a biaxial symmetry [29]. The aminoacidic sequence of the polypeptide chains, rich in glycine and cysteine, is variable at ten positions so that the protein is a mixture of three main isoforms (WGA 1, 2, and 3). Each polypeptide chain contains four hevein domains (carbohydrate-binding domains) indicated as A, B, C, and D. The hevein domains are devoid of a hydrophobic core, but each one contains eight cysteines engaged in disulfide bridges that help stabilize their structure (Figure 1).

The polypeptide chain is resistant to high-temperature exposure, and its conformation undergoes reversible changes in an acidic environment, which is probably related to the presence of many disulfide bridges. The domains A, B, and C bind with high-specificity N-acetylglucosamine residues (e.g., chitin), while the D domain accepts the glycoside aglycones. The C domain can also bind monosaccharides. The protein also has a low affinity for other carbohydrates, and it binds specifically to two types of N-acetylated sugar, N-acetyl-D-glucosamine and N-acetyl-D-neuraminic (sialic acid) [30]. All these structural features contribute to generating a range of biological activities [31]. According to its specificity and structure, the WGA is classified as a chitin-binding lectin composed of hevein domains.

### 2.3. Biological Roles of WGA

Lectins are found in all plant organs, although in quite different amounts according to the function exerted. Storage tissues may contain high amounts of lectins (generally from 0.1 to 10% of the total proteins therein) involved in defence, mainly against fungi and predators [16,17,24]. Other tissues like shoots, roots, leaves, or flowers, on the contrary, contain lectins in very low amounts, even undetectable until induced by stress; these lectins are probably involved in other biological processes related to innate immunity and signal transduction and can fulfil specific functions inside the plant cell or in the interaction with other organisms (e.g., recognition of symbiont species) [17,18,24].

Anti-bacterial, anti-viral, anti-fungal, and especially anti-insect properties of plant lectins, together with their toxicity towards higher animals, have been documented [16,17]. However, in vitro observations must be distinguished from in vivo ones and from observations in a natural environment, and the results must be interpreted with care.

The WGA has been shown to possess anti-fungal and anti-insect properties, which are related to its ability to reversibly bind chitin, the main polysaccharide constituent of the cell wall of fungi and of the exoskeleton of insects [17,32,33].

Specifically, WGA showed clear anti-insect properties in a series of feeding trials with artificial diets [17,34]. For example, WGA showed activity at physiological concentrations against the development of larvae of the cowpea weevil (*Callosobruckus maculatus*) [35]. Furthermore, WGA was lethal to neonate larvae of the European corn borer (*Ostrinia nubilalis*) at low concentrations and was also able to inhibit growth of larvae of the Southern corn rootworm (*Diabrotica undecrmpunctata*) [36]. Also, WGA negatively affected the growth and reproduction of the rice brown planthopper (*Nilaparvata lugens*) [37]. The mechanism of action of the WGA probably involves binding to glycoconjugates in the insect gut (on 3 possible sites: peritrophic membrane, epithelial cells, or digestive enzymes) [17], although it has not been fully elucidated yet. However, the actual capacity of WGA to protect wheat from insects in a field experiment has not been investigated yet [34].

It is possible that WGA also possesses anti-fungal properties [16,17,32,33]. In 1975 it was reported that WGA and other plant lectins inhibited spore germination and hyphal growth of the fungus *Trichoderma viride* [38]; however, some years later it was found that the effect was due rather to the chitinases present as contaminants in the WGA preparation used [17,39]. Another study from the 1970s reported that WGA can bind to the chitin of hyphae and of immature spores of some species of *Penicillum* and *Aspergillus*, slowing their germination, and in the same study the binding of the lectin to the fungi was examined with the aid of its fluorescein isothiocyanate (FITC) conjugated derivatives [40]. In 1999, Ciopraga et al. [41] confirmed that a purified WGA devoid of contamination by chitin inhibited the growth of two species of *Fusarium* in a number of tests on the two *Fusarium* strains and viability tests on potato tuber slices. Again, the ability of WGA to have protective effects in field conditions remains to be studied.

### 2.4. Presence of WGA in Wheat

Mishkind et al. [42] demonstrated that WGA is heterogeneously distributed in wheat embryos and plants. Upon germination, the WGA concentration reduces gradually with maturation. After one month of growth, WGA levels are approximately one-third to one-half of those in ungerminated embryos.

There are not many studies in the literature with reference values for the concentration of wheat germ agglutinin in wheat grains. A study by De Punder et al. [43] on the dietary intake of wheat and other grains presents a table showing the WGA concentration in various wheat-derived products such as wheat germ, semolina, flour, wholemeal flour, raw pasta, cooked pasta, wholemeal pasta, and breakfast cereals, and values range from 0.3 μg/g for cooked pasta to 500 μg/g for wheat germ.

It is likely that the WGA concentration in the wheat grain could vary depending on harvest times, climate, and soil conditions, and cultivars, but there are no systematic studies on these aspects. Peumans and Van Damme [25] reported for chitin-binding lectins in the wheat seed a value of <0.01 g/kg and for the germ 0.1–0.5 g/kg. There are just a few studies in the literature that provide quantitative information of WGA concentration in wheat germ or wheat grains, and these few indications constitute a problem in the utilization of WGA as a marker as, for the time being, there is not a reference value for germ and grains.

As regards the levels of WGA in wholegrain flour, there are a few more studies, however, with different results. This can be explained because commercial wholegrain flours, are in most cases reconstituted by mixing in different proportions different milling streams, and this impacts on WGA concentrations. Studies conducted by Matucci et al. [44] found variable WGA values in wholemeal flour samples from 29.5 (±2.5) to 50.0 (±5.5) μg/g, whereas according to Rojas Tovar et al. [45], commercial wholegrain wheat flour contained 6.6 ± 0.7 μg/g WGA/g. A more recent study detects WGA content in extracts of different samples of wholemeal semolina in the amount of 5.70 (±0.38) μg/g [46].

Table 1 summarizes the values of WGA content in wheat products, obtained by different authors.

## 3. Significance of Lectins and WGA in Human Nutrition

### 3.1. Lectins and WGA Toxicity for Humans

The cytotoxicity of WGA, as of all lectins, depends on the interaction time and affinity with glycoconjugates present on the cell surface. Potential adverse effects due to WGA intake have mainly been studied in animals. In some research, the toxicity of lectins was tested, and it was highlighted that it occurs only in cases of intake in very high concentrations and without any food treatment. Adverse effects usually consist of nausea, bloating, vomiting, and diarrhea. However, these symptoms are extremely rare with normal intake of food prepared for human use [23].

In fact, experimental work carried out in vivo has shown that the toxicity of WGA for the normal gastrointestinal tract occurs at very high concentrations, 7 g WGA/kg body weight taken over a 10-day period, doses higher than those ingested regularly in the human diet [47,48]. On the contrary, concentrations of WGA used in pure form for pharmacological purposes have a more severe effect on human epithelial cells [49].

Due to its specificity for N-acetylglucosamines (GlcNAc) and sialic acids, WGA can bind to the epidermal growth factor receptor (EGFR) or other receptors that have nacetyl-glucosamine in their binding site, such as leptin inhibiting the bond [50].

Additionally, in vitro studies have demonstrated that WGA has an inhibitory effect on nuclear pore complexes (NPCs) depending on the size of the molecule passing through the NPC [51].

In vitro studies have shown that WGA could alter the integrity of the epithelial layer and increase its permeability. Furthermore, WGA influences immune cells by stimulating the synthesis of proinflammatory cytokines. Thus, the biological activity of WGA could have effects on the immune system at the gastrointestinal interface, as better described in one of the next paragraphs [49,52].

Furthermore, in in vitro studies, it has been shown that WGA works directly by stimulating monocytes and macrophages [43]. For example, it has been demonstrated that it can induce the secretion of interleukin 12, which in turn stimulates the transcription and secretion of IFN-γ, the main activating cytokine of macrophages [53]. In the past, it had already been observed that WGA could trigger histamine secretion, inducing NADP-oxidase activity in human neutrophils [54].

Lectins are also involved in autoimmune disease genesis by presenting wrong immune system codes and stimulation of the differentiation of some white blood cells [55,56,57]. Due to the homology between plant and animal lectins, it is likely that the dietary plant lectins, to some extent, can mimic and amplify the effects normally exerted by their lectin counterparts produced by the animals themselves [58].

WGA has effects on activation of the platelets [34], agglutination of red blood cells [59], and increasing the affinity of the insulin receptor and the insulin sensitivity of the cells [60,61,62,63].

### 3.2. Nutritional Significance of Lectins and WGA in Human Diets and Influence of Wheat Processing

Numerous lectins have been isolated from various foods we eat. Lectins are in fact important constituents of our diet [23]. The Mediterranean diet, for example, is a rich source of lectins, and people are thus continually exposed to a wide variety of lectins, which are found in the greatest concentration in raw beans and grains (especially wheat), but they are also found in fruits, vegetables, and nuts [34,64]. WGA is considered the most common food lectin consumed [39]. Lectins are also known in the animal kingdom [58].

In contrast to dietary proteins, most lectins are resistant to degradation by the digestive enzymes and bacteria in the gut [65]. Depending on their structure and biochemical properties, up to 90% of orally administered lectins survive passage through the entire digestive tract in an immunologically intact form, and most dietary lectins act as antinutrients [25]. In animal models, binding to glycoconjugates and glycan receptors of the enterocytes on their luminal surface, lectins were shown to induce alteration of intestinal integrity by compromising nutrient absorption and reducing growth [66,67,68,69].

Raw wheat contains a certain amount of WGA, which may be harmful; however, most of the antinutritional effects of lectins can be eliminated or substantially reduced with proper storage, soaking, germination, and heat treatment such as boiling [70,71].

In wheat, as mentioned above, WGA concentration in the seed is <0.01 g/kg, but data are not available on the mean dietary intake of WGA in humans.

Because WGA is a heat-labile lectin, it is assumed that it will lose its biological activity because of heat exposure, for example, during baking or boiling. In fact, in a normal diet, WGA is not ingested pure but as a component of a complex matrix that is transformed by primary and secondary processing and often cooking into edible foods. The effects of processing and cooking on wheat germ agglutinin (WGA) may vary depending on the method used and the specific conditions applied [23].

WGA is relatively heat stable compared to other proteins when subjected to moderate temperatures; however, prolonged exposure to high temperatures can lead to a significant reduction in WGA activity. For example, studies using wholemeal pasta have shown that the cooking process strongly reduces WGA activity [44]. There is currently no data on the effects of other (thermal) treatments to which cereal products might be subjected, such as baking and frying, but it is expected that effects similar to those that occur during boiling could be observed.

Other manufacturing steps, which do not necessarily involve heat, may also be responsible for reducing the WGA content in foods. Recently, Rojas Tovar and Gänzle [45] documented that fermentation reduces the concentration of WGA in wholemeal doughs. The decrease in activity can depend on many fermentation parameters, such as pH, time, temperature, and specific microorganisms involved.

The germination and sprouting process can lead to biochemical changes, including alterations in protein composition. It is plausible that germination and sprouting may influence WGA levels or activity similarly to other wheat proteins. However, further research is needed to clarify the specific impact of germination on WGA [72].

We must also report that some works have shown that wheat lectins may have potential as a toxic compound and anti-nutritional factor able to induce negative effects on health, even at micromolar concentrations [23,43].

It is interesting to speculate also on the fact that WGA activity decays in a temperature-dependent manner, and this could make WGA a potential indicator of thermal treatment for wheat-derived food products. The effect of processing and cooking on wheat germ agglutinin (WGA) activity is summarized in Table 2.

### 3.3. Impact on Human Gut Health and Immunoregulation

As already mentioned, wheat germ agglutinin (WGA) is a lectin with four specific binding sites for N-acetyl-D-glucosamine and N-acetyl-D-neuraminic acid residues [34]. These ubiquitous sugars constitute key molecular components of membrane glycoconjugates in animal cells. In particular, WGA interacts with the glycocalyx of gastrointestinal cells, and, compared to other plant lectins, it binds to intestinal cell lines of human origin, human colonocytes, and prostate cancer cells at the highest rate [73,74].

In vitro studies suggest that WGA, already at very low concentrations, binds to surface glycans on epithelial cells, causing damage to the base of the villi and thus allowing increased intestinal permeability [23,48,75]. This interaction may facilitate the translocation of both dietary and gut-derived pathogenic antigens to peripheral tissues and activate the immune system. WGA can stimulate the biosynthesis of proinflammatory cytokines. Cytokines, in turn, concur to alter the integrity of the epithelium layer itself. Auto-antibodies can be formed against the tissue, thus resulting in autoimmune disease [49,76,77].

Overall, these findings suggest that WGA can interact with immune cells even in small quantities that escape digestive proteolytic breakdown, are absorbed, and modulate immune cell response to antigens and cause changes in immune cell signalling [29].

WGA has recently been indicated among the compounds that, aside from gluten, may be involved in the pathogenesis of the so-called non-celiac-gluten/wheat sensitivity [78].

Therefore, the action of lectins on the gut is to cause damage, also negatively interfering with the absorption of nutrients through the intestinal wall. We can conclude that they act as “anti-nutrients” with a harmful effect on the intestinal microbiome, negatively modifying the balance of the gut microbiota [79].

This is interesting to underline because there are many studies in the literature that highlight the link between the intestinal microbiota and its interactions with the intestine and the central nervous system. The microbiota-gut-brain axis is able to transmit bidirectional communication, via local, paracrine, and endocrine mechanisms, between the intestine and the central nervous system and connects the emotional and cognitive centers of the brain with the peripheral intestine [80].

Recent advances in research have described the importance of the intestinal microbiota in influencing physiological normality and contributing to the prevention the pathogenesis of neurodegenerative diseases, such as Alzheimer’s and Parkinson’s disease [81,82].

A study by Vojdani et al. [83] has recently discovered a high homology of peptide sequences between a-syn and many food antigens, including wheat lectins, with recombinant monoclonal a-syn antibodies. Alpha-synuclein is a protein that plays a key role in maintaining the production of neurons in the elderly brain [84]. It was observed that these food antigens that resembled a-syn, once passed the mucosal barrier, would aggregate within the enteric cells and then migrate cephalically towards the substantia nigra. But there are still too few studies on this issue, and many more are needed to expand our understanding of these matters.

Considering the recent interest of research on the topic of wheat bran proteins, it might be useful to specify here that several types of proteins are found in the wheat embryo and germ, of which WGA is only a small part. The wheat germ, for example, is rich in globulins (WGG), which represent a class of high-quality proteins that have attracted considerable interest and attention for their distinctive functionalities [85].

The XG Ji et al. study [85] observed the immunoregulatory effects of WGG on immunosuppressed mice induced by cyclophosphamide and explored the immunological activities of WGG. The study highlighted the importance of WGG as an immunomodulator, particularly of its main immune components, the family of wheat germ histones and thermal shock proteins (HSP). These globulins are able to influence cell differentiation Th1/Th2 by controlling the expression levels of mRNA of related genes.

Subsequent studies have observed that in a healthy condition, the intake of wheat germ in the normal diet as an additional food could improve immunity, and as a result, the remodelling of the intestinal microbiota. Its anti-inflammatory ability and changes in the level of cytokines and immunoglobulins have been associated with the gut microbiota. This suggests its possible usefulness as a functional food for strengthening the immune system [86].

A recent study by Fan et al. [87] optimized the extraction and purification of globulin from wheat germ and assessed the molecular weight distribution and structural properties.

However, it is important not to confuse the role and functions of WGA with respect to this large class of globulins, even if we must say that when we eat germ-containing foods, we eat all these different proteins.

### 3.4. Applications of Lectins and WGA in Research and Diagnostics

Thanks to their carbohydrate-binding properties lying at the basis of their biological functionality, lectins are finding interesting applications in crop protection, medicine, biological research, analysis, and even non-biological fields [16,23,29,65,88,89]. Their ability to agglutinate red blood cells has been long used for blood typing. In research, lectins are used to investigate fundamental biological processes like cell proliferation, cell arrest, apoptosis, neoplasmic cell metastasis, leukocyte homing and trafficking, and microbial infection. Lectins are also used to study glycoconjugates in solution and on cells and to characterize and separate cells.

Medical applications of lectins, some of which have reached preclinical and clinical trials, include use as anti-microbial agents (anti-bacterial, anti-fungal, and anti-virus), anti-cancer agents (directly through induction of apoptosis and autophagy, or as advanced drug delivery systems in conjunction with nanoparticles or liposomes), and diagnosis tools for neoplastic cell identification (tumour diagnosis, stage diagnosis, and metastasis detection). Use in drug delivery is being investigated also for other diseases than cancer, e.g., Alzheimer disease. Lectins also find non-medical application in the development of novel techniques such as in a microarray for a high-throughput analysis of glycans and glycoproteins, or even for data-storing techniques, where carbohydrates are used as hardware to code information [23,90].

Specifically, the WGA is being studied for applications in medical and non-medical research, diagnostics and therapy, and even other fields [23,29]. Many examples of these applications can be found in the literature. The ability of WGA to bind to and agglutinate red blood cells has been known for a long time and is used for blood typing. Lectins possessing this ability are usually called haemagglutinins [23,91]. The WGA is also of help in the identification of malignant tumours since tumour cells express altered glycan patterns in their glycoproteins and glycolipids. Such alterations, which are tumour-specific and can be related to the tumour progress and prognosis, can be detected by the binding of lectins to glycoconjugates on the cell surfaces. For example, it was found that WGA and other lectins can bind to different human melanoma cell lines and that the extent of binding to surface-exposed oligosaccharides was related to the ability of the tumor cell to induce metastasis [16]. Thanks to its affinity to glycoconjugates on cell surfaces, WGA can also exhibit direct or cell-mediated cytotoxicity, in a dose-dependent manner, resulting in necrosis or apoptosis according to the cell line used in the test; different cell lines, including murine and human tumor cell lines, were successfully tested [92]. Another application is related to the fact that WGA, conjugated with nanoparticles, liposomes, or other forms of functionalization, enhances oral delivery of insulin [16,93] and acts as a carrier of chemotherapeutic agents to specifically target tumour cells [16]. For example, Neutsch et al. [94] showed that the binding affinity of nanocarriers to urothelial cells increased when they were functionalized with WGA. These microparticles also showed enhanced anti-neoplastic activity: WGA functionalised nanoparticles showed increased absorption and were able to more effectively deliver thymopentin to the intestinal mucosa of Wistar rats [95]. WGA strongly interacted with cells of the human urinary carcinoma 5673 and was used to target bladder cancer cells [96]. Furthermore, the WGA conjugated carrying agents can more easily overcome the main physiological barriers, including the blood-brain barrier or the urothelial barrier [29,94]. This is particularly important because it could open new ways to effectively deliver drugs for, e.g., Alzheimer’s disease. A recombinant WGA linked to the effector Fc region of murine IgG2a was designed for mycosis treatment and demonstrated activity towards a number of fungi, from mice pathogens to human pathogens like *Candida albicans* [29]. This might open the way also to crop protection treatments. WGA-functionalised silver nanoparticles were used to detect specific Gram-positive and Gram-negative bacteria. WGA was used to improve the extraction and purification of erythropoietin and darbepoetin, two kinds of recombinant proteins [97]. The above-mentioned applications are summarized in Table 3.

## 4. Extraction and Quantification of WGA in Whole-Grain Wheat-Derived Products: Data from the Literature and Experiences with Available Rapid ELISA Kits

### 4.1. Chemical Methods for the Isolation, Purification, Determination, and Quantification of WGA in Wheat and Its Products

The procedures for the isolation, purification, and crystallization of WGA have been described in the literature since the 1970s. Nagata et Burger published a paper in 1972 [98] that concerned the isolation and crystallization of WGA, as well as its physical and chemical properties. Subsequently, the same authors developed a new purification procedure starting from unprocessed wheat germ [99]. Other studies related to the purification of wheat lectin followed over the years [100,101].

Proteins can be studied by means of different analytical techniques, which can be more or less expensive and time-consuming. Measurements based on biological activity, such as enzyme-linked immunosorbent assays (ELISA), are highly sensible and specific, generally rapid, and low unit cost. The strong and very specific antigen-antibody interaction has therefore been used in a great number of applications in the food area, and many rapid kits are commercially available [46].

Following this trend, in 2002 Vincenzi et al. [13] developed an immunoenzymatic method for the quantitative determination of dietary lectin activities employing immobilized glycoprotein. The proposed method allowed a quantitative determination of the wheat lectin activity starting from a simple dilution of the extracts from the raw sample. One g of wheat germ was homogenised in 10 mL of phosphate buffered saline (PBS) solution, and the obtained suspension was stirred overnight at 4 °C. The homogenate was centrifuged at 9000× *g* at 4 °C for 15 min, and the supernatant was clarified by filtration on Whatman No. 4 paper. The resulting solution was diluted in PBS-bovine serum albumin (BSA) in ratios varying from 1:100 to 1:1000. Subsequently, the sample was analysed by the ELISA method with specific antibodies for lectin. To set up the method, glycoproteins immobilized on the microtiter plate were used. The following parameters were considered: glycoprotein type, glycoprotein concentration, and the pH of the coating buffer. The best conditions in terms of precision, detectability, and sensitivity have been identified for coating the plates with ovalbumin, which, as demonstrated by the literature, is in fact rich in GlcNAc (N-acetyl-D-glucosamine) [102]. The optimal concentration for coating was found to be 20 mg/mL and using buffer 50 mM Na-carbonate (pH 9.6). Under this condition, ELISA responses were dose-dependent for WGA (range 30–1000 ng/mL). Linearity of dose/response curve was obtained in the concentration range 125–500 ng/mL for WGA [13].

The results of the experiments conducted on the activity of the WGA found by ELISA in the wheat germ extracts were in good agreement with data in the literature [103] as well as with data obtained in the standard agglutination test [13]. The following year, using the previously described ELISA method, the same authors published another article to determine the concentration of WGA present in raw and cooked wheat-derived foodstuffs [44]. Detectable amounts of WGA were found in raw foods and in wheat flours, while variable quantities of agglutinins were found in wholemeal pasta, probably because of thermal inactivation during food processing.

The thermal gradient gel electrophoresis (TGGE) technique was therefore applied to analyze the thermal stability of WGA and it was observed that the biological activity of WGA decreased as a function of heating temperatures and exposure time in an S-shaped fashion with an inflection point around 65 °C. The authors therefore declared that the WGA could represent a biochemical “indicator” that allows to determine the heat treatment to which wheat-derived foods are subjected during processing.

In 2007, Güll et al. [104] developed a new ELISA protocol for the determination and quantification of wheat germ agglutinin. A sandwich ELISA based on the capture of lectin in wells coated with PGM (porcine gastric mucin) and detection of bound WGA by a first lectin-specific antibody followed by a second peroxidase-labelled antibody was created. The optimized protocol allowed quantification in the range 10 to 1000 ng/mL WGA with a coefficient of determination of 0.9991. Subsequently, in 2009, the ELISA method by Vincenzi et al. [13] was modified for a study concerning biochemical markers for the evaluation of proportions of wheat-grain tissues in milling fractions [14]. WGA was extracted from different fractions of wheat grains obtained by peeling, pearling, and milling steps. After extraction with dilute hydrochloric acid, samples were serially diluted with PBS containing BSA. A commercial WGA was used for the preparation of the calibration standards (range 0.2 to 125 ng/mL). Ovalbumin in PBS was used to coat the flat bottom microtiter plates. The dishes were incubated with purified anti-rabbit immunoglobulin conjugated with alkaline phosphatase. After one last washing, the plates were incubated with the enzyme substrate. The enzymatic reaction was stopped by the addition of sodium hydroxide to the wells. Absorbances were read at 450 nm. The authors concluded that WGA seems to be a promising marker of wheat germ even if the quantification method was not able to quantify the germ proportion in the milling fractions.

In 2012, Baieli et al. [105] developed an efficient affinity chromatographic matrix based on chitosan for WGA purification. Different matrices consisting of minispheres of chitosan crosslinked with epichlorohydrin were used, and the best response of high purity degree (95%) occurred with minispheres crosslinked with 250 mM epichlorohydrin at pH 8.5. Older studies [106] relied on the specificity of WGA for the GlcNAc residues of chitin and chitosan, obtaining yields of 70–80% with successive purification cycles by an affinity flocculation process with dissolved chitosan (20 mg/mL). This process has a high yield, but the separation between chitosan and WGA requires a size exclusion separation. Zeng and Ruckenstein in 1999 [107] have instead obtained a lower result (41%) for the complete purification process, using chitin porous membranes, observing a large loss of WGA in the diafiltration steps of the extract.

Studies by Rojas Tovar et al. in 2021 [45] explored the fate of WGA during sourdough fermentation of doughs prepared with whole-grain flour. WGA was extracted based on the protocol of Baieli et al. [105] from two wholemeal flours. The quantification of WGA present with and without sourdough fermentation was carried out using an ELISA kit obtained from MyBioSource (San Diego, CA, USA), and the test was performed following the supplier’s instructions. After adding of samples (50 µL), 100 µL of horseradish peroxidase (HRP) conjugate was added, and the plates were incubated for 60 min at 37 °C. After incubation, the wells were washed four times with washing solution, and 50 µL each of chromogenic solutions A and B were added. The plates were incubated in the dark at 37 °C for 15 min; 50 µL of stop solution was added, and the absorbance was determined at 450 nm. ELISA quantification of WGA demonstrated that sourdough fermentation reduces the concentration of WGA in whole wheat doughs.

An interesting study was conducted in 2022 by Marengo et al. [46] on the quantification of protein “biomarkers” in wheat-based foods (including WGA), which can represent suitable indicators for assessing the presence/absence of specific food ingredients or the type and intensity of food processes. The study focused on the design of the procedure for the recovery of the protein biomarkers in a form suitable for reliable identification and quantification and on the critical analysis of the difficulties associated with the plain transfer of an analytical protocol from one product to another. A rapid ELISA approach was selected for quantification of WGA in extracts obtained by diluted solutions of acids or bases. WGA was initially isolated from various matrices (wholemeal semolina, refined semolina, fractions of fine and coarse grinding of durum wheat, and wholemeal pasta) by extraction with HCl in the presence of a reducing agent and further purified by precipitation with 35% ammonium sulfate and ion exchange chromatography. The same samples were analyzed with different procedures such as Western blotting, SDS-PAGE, and LC-MS/MS to confirm the presence of WGA-derived peptides. After the detection of WGA in the extracts from whole-grain milling fractions, an indirect competitive ELISA was used for its quantification. The authors reported a value of 5.70 ± 0.38 μg/g for wholegrain semolina and <0.025 μg/g for refined semolina and stated that at least 90% of the WGA expected to be present in the durum wheat kernel could be determined by ELISA in the acid-extracted material.

However, when the same protocol was applied to wholegrain pasta samples, WGA was not detected. It was possible to detect WGA in pasta by replacing the acid extraction medium originally used by Nagata and Burger [99] with 50 mM NaOH. With this procedure and ELISA, it was possible to quantify WGA in wholegrain pasta and to notice a decrease in the WGA amount of pasta compared to the unprocessed raw material. This behaviour is certainly due to the changes induced by pasta processing on the protein network (made up by gluten and other proteins) that may entrap WGA, preventing its release and quantification.

This study underlines the importance of how much physical treatments can influence the recovery of a protein analyte in a non-linear way depending on multiple interactions acting simultaneously, and therefore there is a need to design improvements that consider the specific situation in terms of ingredients and their ratios, as well as the nature and intensity of treatments used in the processing steps.

### 4.2. Experiences with Rapid Test Kits Available on the Market

Following the examination of the methods present in the literature, we wanted to test in our laboratory the performance of two different commercially available rapid ELISA kits for the quantification of WGA in wheat germ and wholemeal flour samples. The tested samples were wheat germ obtained from an industrial mill, commercial bran, and whole-grain flour produced in our laboratory by means of a Bühler MLI 204 laboratory mill from soft wheat grains.

The first WGA ELISA kit was obtained from MyBioSource (San Diego, CA, USA), and the test was performed following the supplier’s instructions. This quantitative sandwich ELISA is indicated as specific for the determination of WGA levels in undiluted original plant, body fluids, and tissue homogenates. The detection range of this kit is 0.25 ng/mL–8 ng/mL. Two samples were analysed, one of commercial bran and one of wholegrain wheat flour. One g of sample was homogenized with 10 mL of PBS; the homogenate was centrifuged at 3000 rpm for 20 min, and subsequently the supernatant was used for the test. Unfortunately, the results obtained with this kit were not reliable, and for this reason they are not reported. The problem observed was that we did not obtain results that were in any way comparable with data in the literature for similar products, although in line with the reference value for Chinese wheat germ that was provided by the producer of the ELISA kit.

The other kit used for detecting WGA was purchased from Novatein Biosciences (Woburn, MA, USA). The kit is designed to quantitatively detect the levels of WGA in solution, body fluid, and wheat germ extract. The WGA ELISA test is based on the competitive enzyme immunoassay principle and involves the use of biotinylated WGA as a tracer. Two different procedures were followed for the extraction: (1) 1 g sample in 20 mL of RIPA buffer as in the kit instructions, and (2) 1 g sample in 5 mL of RIPA buffer, as suggested by the product specialist we contacted. Both procedures continued as suggested by the specialist: extraction in a 40 ℃ water bath, stirring constantly, for 4 h. After that, ultrasonic bath + Ultraturrax homogenizer for 10 × 10 s cycles with medium power on ice. Centrifuge at 15,000× *g* for 15 min to get the clear supernatant. Finally, dilution 10-fold before the ELISA assay. We found some problems with procedure (2). The amount of sample was too much with respect to the amount of buffer, so we obtained a dense slurry, and the extract after centrifugation was not perfectly clear. The results obtained this time were within the range present in the literature but cannot be considered reliable and accurate. With procedure 2, we obtained much lower values than with procedure 1, whereas we were expecting comparable results: for the wheat germ, with procedure 1, we obtained a mean value of 759 (±85) μg/g (triplicates); instead, with procedure 2, we obtained a WGA level of 160 (±15) μg/g. For wholegrain wheat flour, we obtained values more like each other with both procedures and close to those detected by Matucci et al. [44]. In fact, with procedure 1, the mean result was 73 (±0.9) μg/g, and with procedure 2, the mean value was 55 (±2.5) μg/g.

From our practical experience, we therefore concluded that the commercially available rapid kits for the detection and quantification of WGA, even if the recommended extraction procedure is accurately followed, are not yet useful for the reliable quantification of WGA in whole-grain wheat raw materials let alone thermally processed products.

**Figure 2:**
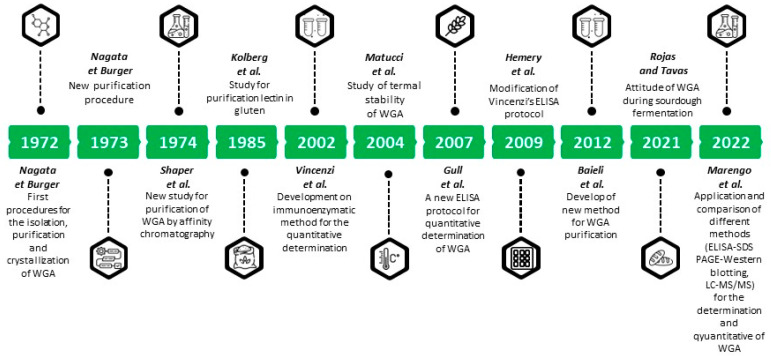
Timeline of studies of WGA as marker of whole-grain status in wheat foodstuff [13,14,44,45,46,100,101,102,103,106,107].

In order to graphically summarize the content of this chapter, a timeline of the various historical steps in the analytical determination of WGA is reported in Figure 2.

## 5. Conclusions

From all we have said, we can conclude that WGA is an interesting and promising molecule in different fields, and new applications are experimented with and reported.

As regards the possibility of WGA to be used as a marker to assess the whole-grain status of wheat raw materials and foodstuffs or the presence of germ tissue in foodstuffs, if it is true that its detection is an indicator of the presence of the germ tissues in a given sample, it must be considered that its extraction and quantification are not yet standardised and different methods give different results that are not comparable with each other and, moreover, rapid methods based on ELISA, which make use of commercially available kits, also according to our experience, so far, do not give repeatable, reproducible, and therefore reliable results with wheat materials.

Also, considering WGA as a biological marker of “wholegraininess,” to evaluate an unknown wheat ingredient and classify it, it would be useful to have an idea of the range of WGA values that can be found in wheat kernels considering varieties and growing conditions to compare results. A whole-grain flour, according to the above-reported accepted definitions, should in fact have the same WGA amount as the wheat grain. Unfortunately, these reference data are totally lacking, and research in this respect should be encouraged.

The assessment of the whole-grain status for a whole-grain food is also very important for consumers and industry alike. In wheat-based foods, ingredients can undergo processing, which may change WGA structure and extractability; some authors reported that pasta making influences the protein network (made up of gluten and other proteins) that may entrap WGA, preventing its release and quantification.

Moreover, the study of a protein by means of immunochemical approaches can be problematic in food matrices where several proteins are present, which can be linked in a network or physically entrapped in a process-generated structure, as it happens in cereal-based products such as bread and pasta. In these situations, for the quantification of a specific protein, it is necessary to break the network, but the protein under question must still be recognizable.

From a nutritional point of view, it would be useful to have an estimate of the intake of WGA with the diet to clarify its effects on human health.

Therefore, the topic of the objective recognition of the whole-grain status of a foodstuff continues to be a hot one in cereal science and technology, and WGA cannot be considered, as for now, a reliable marker for the determination of this status in wheat and its products. In a recent study by Di Stasio et al. [108], a proteomic analysis of *Triticum durum* germ was performed using shotgun proteomics, and more than 900 of the 1168 identified proteins were annotated. The authors concluded that the identification of abundantly expressed proteins, according to the iBAQ value, can enable the research community to develop efficient proteomics-based techniques, an alternative to ELISA, for the identification of whole-grain products.

It is also noteworthy to say here that besides the topic of objective recognition of whole-grain status in wheat-based foods, the utilization of cereal waste (bran) for the production of bioactive ingredients from bran proteins to be used in the manufacturing of functional foods [109] certainly calls for the need of better knowledge in general and standardised analytical tools and procedures for the analysis of germ proteins, including WGA.

## Figures and Tables

**Table 1:** WGA content in germ and wholemeal flour found by different authors.

Material	WGA Content	Method	Reference
Wheat seed Wheat germ	<0.01 g/kg 0.1–0.5 g/kg	not specified	*Peumans and Van Damme, 1996* [25]
Whole wheat flour	6.6 ± 0.7 µg/g	ELISA	Rojas Tovar and Gänzle [45]
Wholemeal flour	50.0 ± 5.5 and 29.5 ± 2.5 µg/g	ELISA	Matucci et al., 2004 [44]
Wheat germ Wholemeal flour	from 6 to 8 µg/g 5.70 ± 0.38 µg/g	LC-MS/MS ELISA	Marengo et al., 2022 [46]

**Table 2:** Influence of processing and cooking of wheat food ingredients and foods on the biological activity of wheat germ agglutinin (WGA).

Type of Treatment		Product	Effect on WGA Activity	Reference
Thermal	boiling	wholemeal pasta	strongly reduces	Matucci et al., 2004 [44]
Non thermal	fermentation	wholemeal flour	reduces	Rojas Tovar and Gänzle. 2021 [45]
	germination, sprouting	wholemeal flour	reduces	Adamcová et al., 2021 [70] Pryme and Aarra., 2021 [71] Gunathunga et al., 2024 [72]

**Table 3:** Applications of WGA in research and diagnostics.

Application	WGA Activity	Reference
Blood typing	Red blood cells agglutination/aggregation	Van Buul et al., 2014 [23] Liu et al., 2020 [91]
Identification of malignant tumours	Direct contact, adhesion, binding to cell membrane or receptors	Mishra et al., 2019 [16]
Cancer treatment	Cytotoxic effects resulting in apoptosis or necrosis on diverse cancer cell lines	Ryva at al., 2019 [92]
Advanced drug delivery	In conjunction with nanoparticles, liposomes or other forms of functionalization	Mishra et al., 2019 [16]; Wood et al., 2008 [93]; Balčiūnaitė-Murzienė et al., 2021 [29]; Kuo et al., 2017 [90]; Neutsch et al., 2013 [94]; Yin et al., 2006 [95]; Plattner et al., 2008 [96]
Anti-fungal treatments	Recombinant WGA-Fc inhibits the fungal growth	Balčiūnaitė-Murzienė et al., 2021 [29]
Detection of Gram-positive and Gram-negative bacteria	WGA-functionalised silver nanoparticles	Balčiūnaitė-Murzienė et al., 2021 [29]
Improvement of extraction and purification of erythropoietin and darbepoetin (human recombinant glycoproteins) from equine plasma	Pre-treatment with wheat germ agglutinin immobilized on Sepharose gel	Stanley and Chua, 2014 [97]

## Data Availability

No new data were created or analyzed in this study. Data sharing is not applicable to this article.
